# Supplementary material for: Implementing in-situ self-organizing maps with memristor crossbar arrays for data mining and optimization
Source: Nat Commun. 2022 Apr 28;13:2289. doi: 10.1038/s41467-022-29411-4 (PMC9051161; doi:10.1038/s41467-022-29411-4)
Supplement: Supplementary file 1 — Supplementary Information [file 41467_2022_29411_MOESM1_ESM.pdf]

Implementing *in-situ* Self-organizing Maps with Memristor Crossbar Arrays for Data Mining and Optimization

Wang et al.

Supplementary Figures 1-11.

Supplementary Note 1.

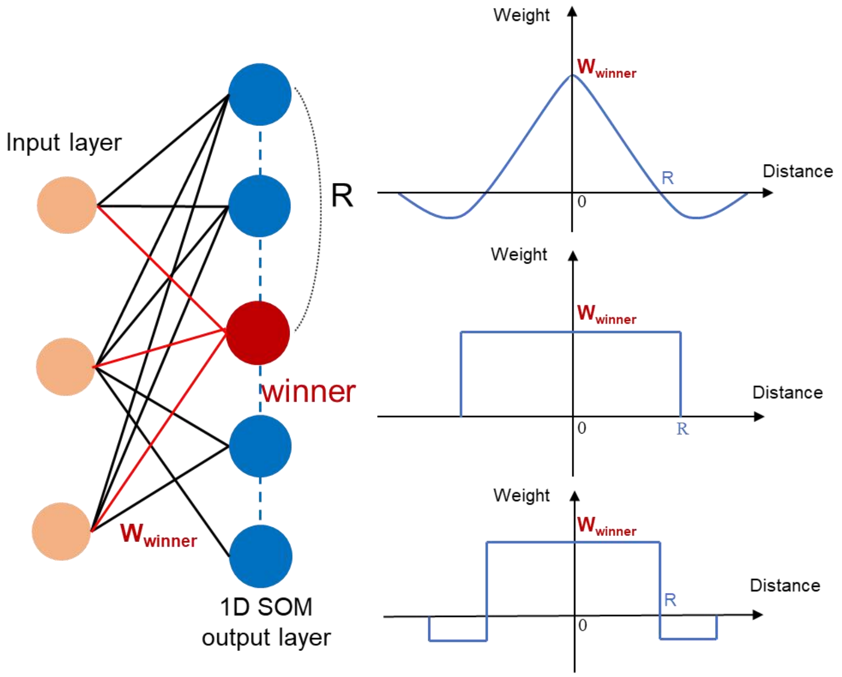

Supplementary Figure 1. Schematic of the 1D SOM. SOM is an unsupervised network including one input layer, one output layer, and the fully connected synapse matrix. During training, not only the winner's weights (red) but the weights of other neurons (blue) are also updated through the neighborhood function. Examples of three common neighborhood functions in SOMs are Gaussian function, bubble function, and Mexican hat function (R is the margin of the winner area).

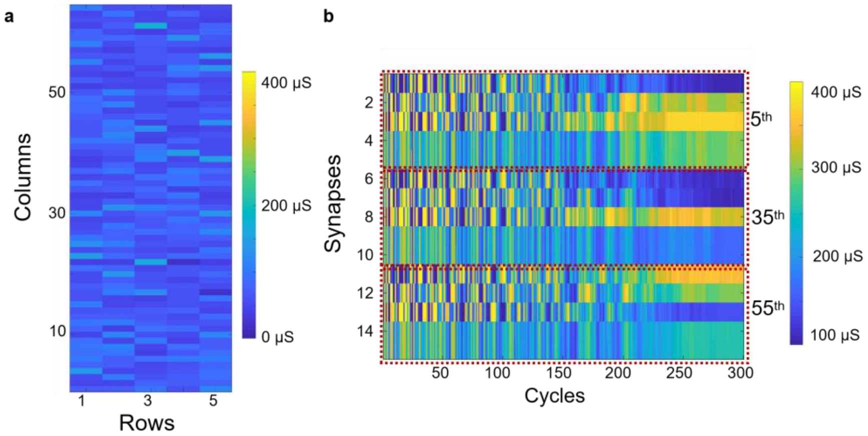

Supplementary Figure 2. Weight map of the color mapping application. (a) The weight map of the selected memristor array before training. All the devices are initialized to HRS (around 50 µS). (b) Evolution of the device conductance in three columns (the 5th, 35th, 55th column) during the training process.

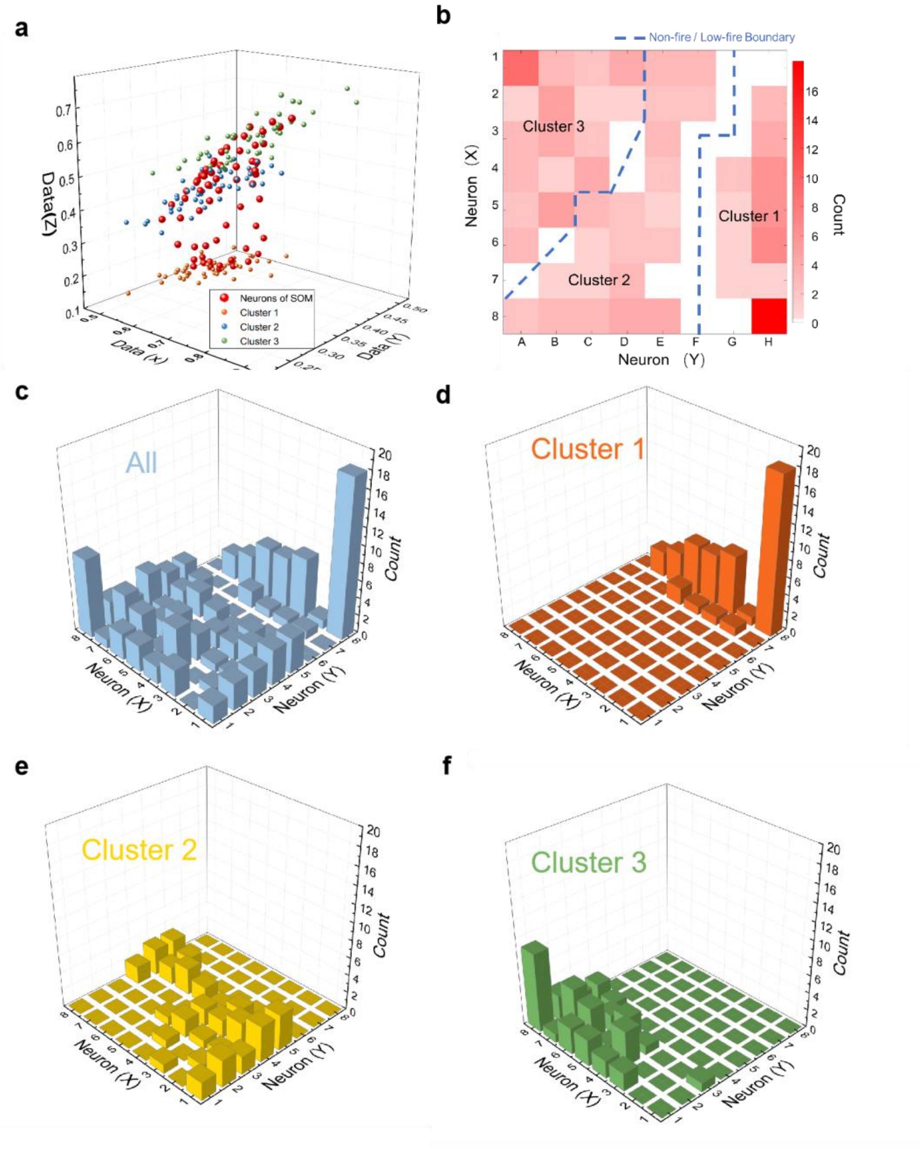

Supplementary Figure 3. **The clustering ability of our SOMs tested by IRIS dataset.** The clustering ability of our SOM is proven by 5 (3 data rows and 2 squire rows) × 64 memristor array with IRIS dataset (150 points from each of the three iris flower species: Setosa, Virginica, and Versicolor with four different features). Here only three features are used in clustering. (a) Weight map of the SOM after training in features space. The big red dots represent the neurons' location of the SOM, and the small orange, blue and green dots are three different kinds of flowers, respectively. (b) Clustering result after training process A 'non-firing or 'low-firing' boundary can be found between different clusters. Three clusters can be defined by the 'non-firing’ or 'low-firing' boundary. (c) The frequency of neurons' response with all data points. (d)~(f). The frequency of neurons' response with different clusters. For the data in the same cluster, they always fire the neurons in the same region. And after the training process, the memristor-based SOM system enables 94.6% high classification accuracy.

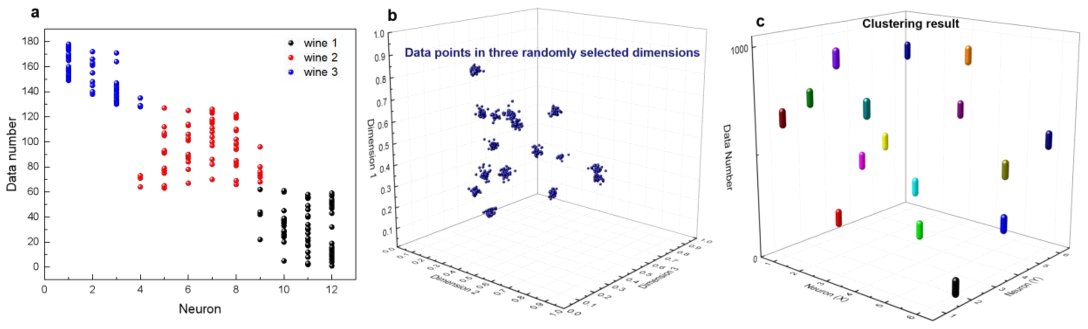

Supplementary Figure 4. **The clustering ability of our SOMs tested by other datasets.** (a) The clustering ability of the 1D-SOM is proven by 22 (13 data rows and 9 squire rows) × 12 memristor array with wine dataset (178 points, three kinds of wine). The most common unsupervised clustering algorithm is the K-mean clustering, which needs to know the number of clusters. We cannot directly classify the three kinds of wine for our SOM, but we can cluster the wines into 12 different clusters. And because of the neighborhood function, we can see the similarity of the wine. And the same kind of wine is close to each other. 95% accuracy can be achieved if the neurons are equally divided into three clusters. (b) Gaussian distribution data set (1024 points, 16 different clusters, 32 dimensions) in three selected dimensions. (c)The perfect result to show the clustering ability of our 2D SOM. A 48 (36 data rows and 12 squire rows) ×64 (8×8 2D SOM) crossbar array with randomly initialized conductance is used for the training. 16 different clusters have been successfully tested.

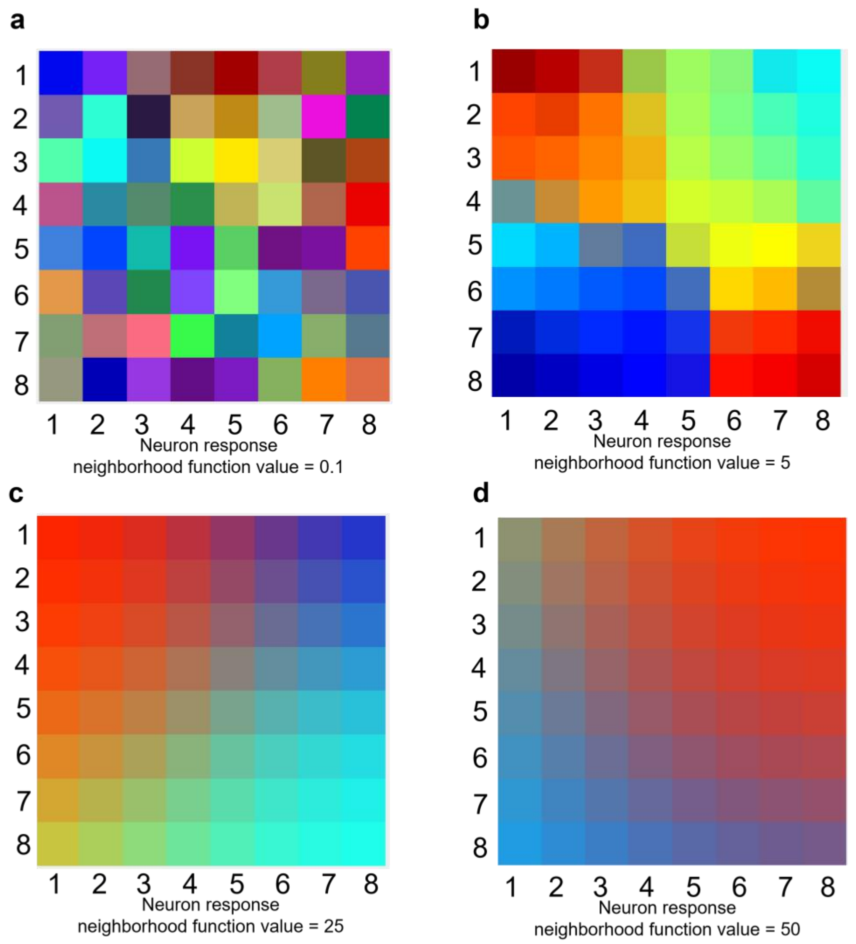

Supplementary Figure 5. Influence of neighborhood function factor for color mapping and clustering. (a), (b), (c) After 600 training epochs neuron responses when the input color is white (1,1,1) with different neighborhood function value (a) 0.5, (b) 5, (c) 50, the three weights of each neuron are the RGB elements of the color. When the neighborhood function is very small, the connection between the winner and neighborhoods is negligible. As a result, SOMs will lose the ability to recognize mixed colors from neighborhood function, and there is no obvious topological relationship of the neuron response. With the increasing neighborhood function, the SOMs show better clustering ability. When the neighborhood function becomes enormous, the connection between the winner and the neighborhoods will be so strong that the total number of the clusters will be decreased during the test process. Based on this principle, a dynamic compressed rate can be achieved just by tuning the neighborhood function factor.

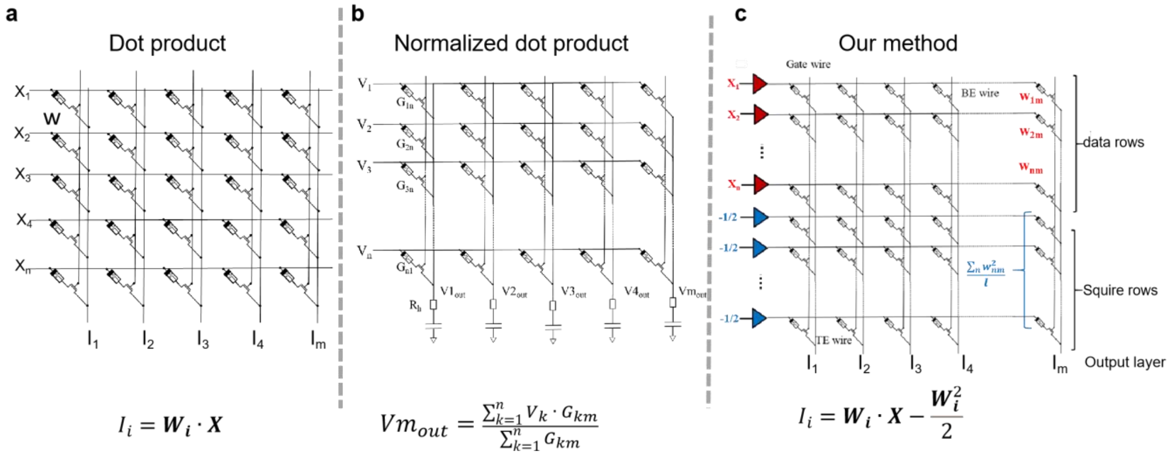

Supplementary Figure 6. Schematic of memristor crossbar array for similarities calculation. (a) Dot product method (b) normalized dot product [1]. Besides the Euclidean distance, the cosine similarity is a popular metric for similarity measure between vectors **X** and **Y**, which can be calculated by **XY**/(||**X**||·||**Y**||). The normalized dot product approximates the cosine similarity by dividing the dot product by the l-1 norm of Y instead of the l-2 norm. The conductance of the memristor can be acted as the weight of the neuron. As Rh is a high resistance, when a series pulses are applied as the input, for each column, the output is approximately the normalized dot product of the inputs and weights and can be determined by $Vm_{out} = \frac{\sum_{k=1}^{n} V_k \cdot G_{km}}{\sum_{k=1}^{n} G_{km}}$). Compared to the dot product, the normalized dot product shows a rougher similarity estimate. However, the Euclidean distance we measured based on our squire method presents higher accuracy than the normalized dot product. (c) Schematic of our method.

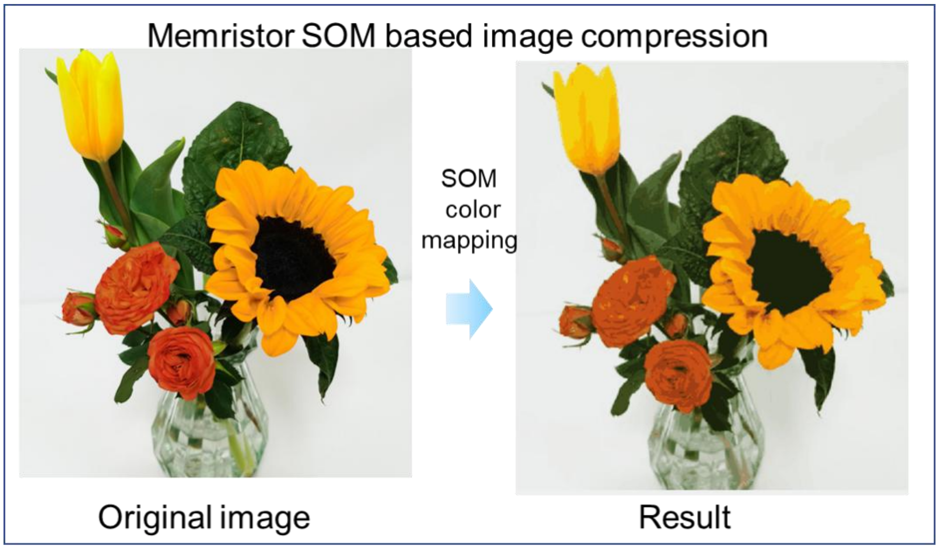

Supplementary Figure 7. Result of SOM-based image compression. Shows the result of image compression implemented with the hardware memristor SOM system. The original image (left panel) is processed, the original image pixels are applied into the memristive SOMs, and the data row weight vector of the winner is the RGB value of the new compressed image pixel. Image is successfully compressed with acceptable resolution.

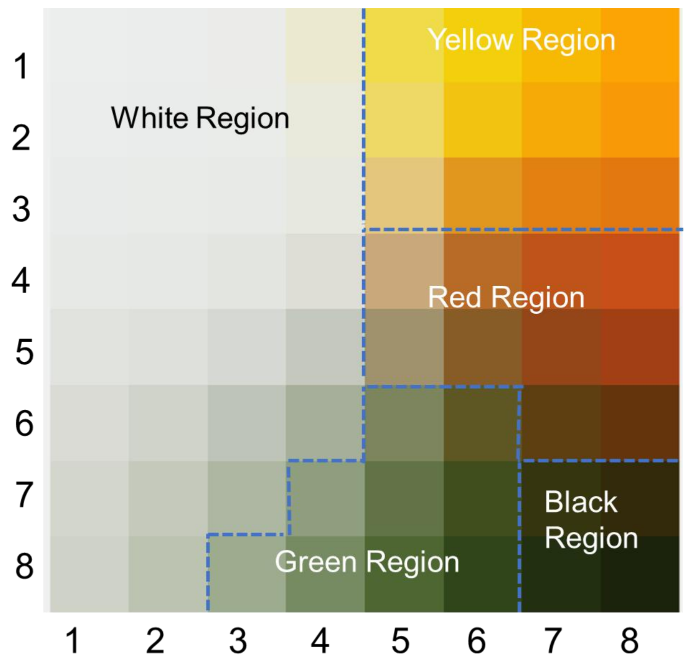

Supplementary Figure 8. Output neuron response for image segmentation. The neurons in the output layer are clustered into five categories according to each neuron's position and response color. Here five different clusters can be obtained.

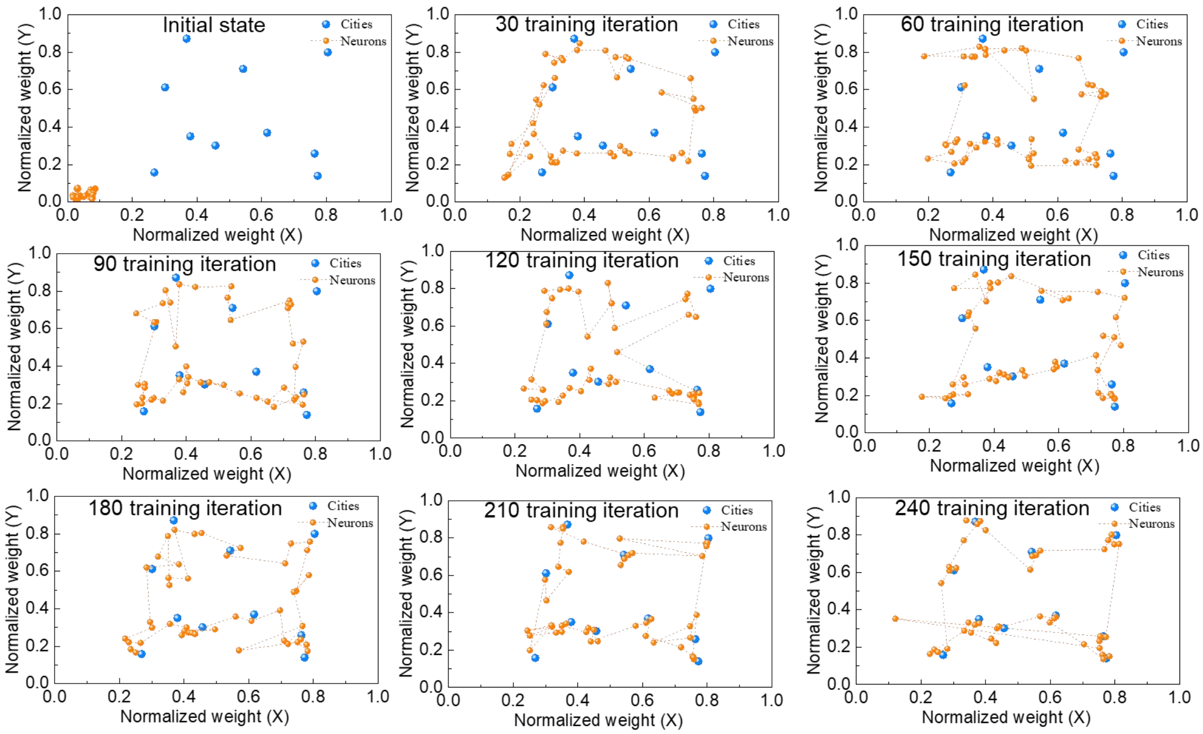

Supplementary Figure 9. A 10 cites TSP by 4×45 crossbar array, normalized weight in city space form initial state to 240 training iterations in the training process. (Experimental) The black dots represent the position of the cities; orange dots are the normalized weights of 45 different neurons, and the red dashed line is the possible route determined by the sequence of the neurons. At the initial states, all the devices are in the HRS. During the training process, the winner node moves in the city plane and induces its neighborhood on the ring to do so, but with a decreasing intensity along the ring. At the beginning of the training process, most nodes tend to converge together with a larger neighborhood function. With a gradually decreasing neighborhood function in the training process, the nodes will progressively become independent and eventually attach to different cities. Finally, the neighborhood function will only affect the winner itself, and the shortest route will be found. Due to the writing error and the variation of the device, some synapses are offset the original positions. But the extra neurons help to overcome this issue and will not affect the result.

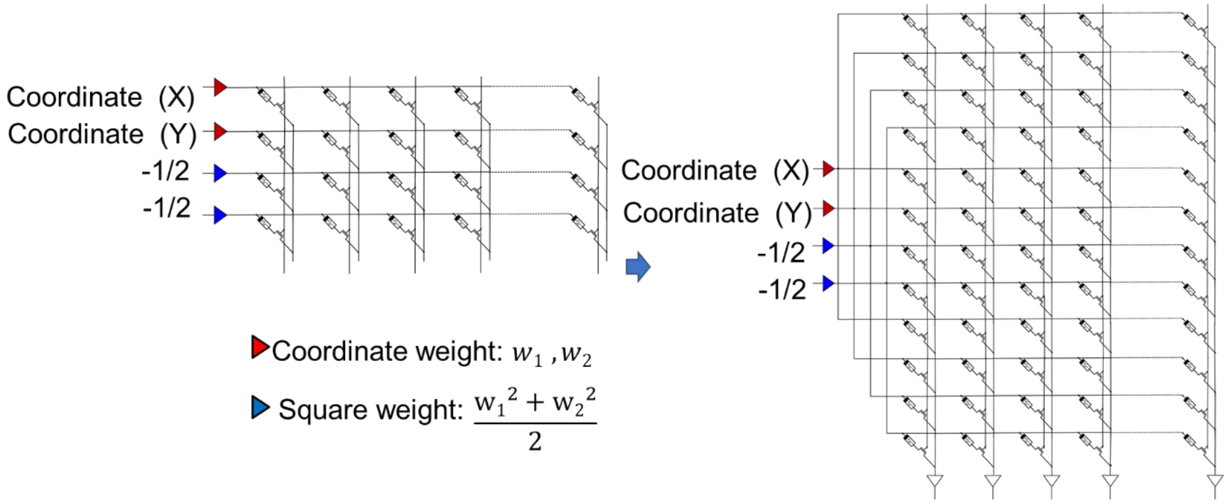

Supplementary Figure 10. Schematic of multiple devices as one weight. A simple method has been adopted to overcome the serious impact of the writing error. In our system, we do not need a complex design. Due to the intrinsic structure of the SOMs (low dimensional inputs), additional rows can be directly used to act as multiple devices for one weight. For example, the original SOM is implemented by a $4\times N$ array. A $12 \times N$ array, which can be divide into three identical $4\times N$ sub-arrays, is adopted to implement 3 devices as one weight SOM. And the inputs of all the sub-arrays are the same so that they can share the same input channels. With this method, we only need extra area costs in the memristor array part. No extra circuits such as DAC and ADC are needed.

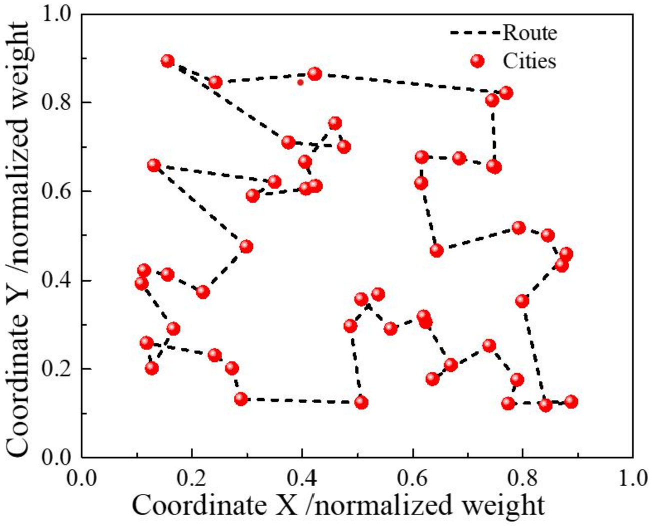

Supplementary Figure 11. **A larger-scale TSP simulation.** Simulation result for 50 city TSP by our memristor-based SOM with the ideal device. To show the potential of solving the complex problem of our system, a 50-city TSP based on our SOMs has been tested. The red dots represent the position of the cities. After training, the normalized weight vectors of nodes are mapped into the city space; the neurons are converted into different cities. The black dashed line is the shortest route determined by the sequence of these neurons in 3D space. This result proves that not only 2D TSP but our memristor-based SOM also can solve more complex optimization tasks.

Supplementary Note 1: Performance analysis of the memristor-based SOM.

We present simple estimations of the performance for two different tasks. 1) Images compressed/segment with a trained SOM. 2) Solving ten-city TSP with a memristor-based SOM.

Table 1. Parameters of two different applications

Application	Array size	Weight	Training epochs	Inference times
Image compressed/segment	5×64	320	~	Image size (500 ×600)
10 city TSP	12×45	540	210	10

Energy estimation of task 1:

With a trained SOM, we only need an inference process to implement image compression and segment.

In the inference or reading process, the maximum inference voltage is 0.2 V, the voltage width is 10 ns, and the average conductance is around 10 kΩ. The Joule heat dissipated by a single memristor is around

$$E_{m_{read}} = 10ns \times \frac{0.2V^2}{10k\Omega} = 40\ fJ$$

The energy consumption in the memristor array for compressing or segmenting a 500×600 size image is

$$E_{m_{test}} = E_{m_{read}} \times n_{weight} \times size_{image} = 40\ fJ \times 320 \times 500 \times 600 = 3.84\ \mu J$$

Energy estimation of task 2:

In the inference or reading process, the maximum inference voltage is 0.2 V, the voltage width is 10 ns, and the average conductance is around 10 kΩ. The Joule heat dissipated by a single memristor is around:

$$E_{m_{read}} = 10ns \times \frac{0.2V^2}{10k\Omega} = 40fJ$$

So the energy cost of the whole array in the inference process is:

$$E_{mtest} = E_{m_{read}} \times n_{weight} \times n_{cities} = 40fJ \times 540 \times 10 = 216pJ$$

Considering adding the external circuit part. The maximum intrinsic energy cost of a single DAC[2], ADC[3], and TIA[4], could be estimated as

$$E_{DAC} = 25mW \times 10ns = 250pJ \quad \text{(12-bit, 250MHz sampling rate)}$$

$$E_{TIA} = 0.2mW \times 10ns = 2pJ$$

$$E_{ADC} = 1.26mWs \times 10ns = 12.6pJ \quad \text{(6-bit, 1GHz)}$$

In addition, the 45 output neurons could have their TIA signals converted to digits with $n_{ADC} = ceil\left(\frac{n_{output}}{10ns \times 1GHz}\right) = 5$ 6-bit ADCs converting at 1GHz. The total energy cost of the test process is

$$E_{test} = E_{mtest} + n_{cities} \times (n_{DAC} \times E_{DAC} + n_{TIA} \times E_{TIA} + n_{ADC} \times E_{ADC})$$
$$= 216pJ + 10 \times (250pJ \times 540 + 2pJ \times 45 + 12.6pJ \times 5) = 1.38\mu J$$

In the training process, the maximum writing voltage is around 2.2V. 2.2V 5ns pulses could program the memristor used in this study.

$$E_{m\_write} = 5ns \times \frac{2.2V^2}{10k\Omega} = 2.42pJ.$$

Here we assumed that the neighborhood function is constant and affects half of the array. And with the write and verify scheme, each write process needs 50 verification steps.

$$E_{write} = E_{m\_write} \times 0.5 \times n_{weight} \times n_{verify} \times n_{training} = 2.42pJ \times 0.5 \times 540 \times 210 \times 50 = 12.71nJ$$

Considering adding the external circuit part. DACs shall produce the transistor gate voltages during programming. The total energy cost of the training process is

$$E_{train} = E_{write} + n_{verify} \times n_{training} \times n_{weight} \times E_{DAC_{write}}$$

$$=12.71nJ + 50 \times 210 \times 540 \times 25mW \times 5ns = 0.71\ mJ$$

Comparison with the SOM in CMOS platform:

The inferencing dynamic power consumption of the memristor array part of a 5×64 SOM at a clock frequency of 200MHz is around $40\ fJ \times 5 \times 64 \times 200MHz = 2.56\ mW$.

The one-shot updating dynamic power consumption of the memristor array part of a 5×64 SOM at a clock frequency of 200MHz is around $2.42\ pJ \times 5 \times 64 \times 200MHz = 154.mm\ mW$.

For our memristor array, the maximum writing voltage is around 2.2V. 2.2V 5ns pulses could program the memristor used in this study. The frequency is around 200MHz. Due to the 128×64 size memristor array, our hardware system can implement a SOM with 8×8 map size and 128 vector dimensions. As a result, the ideal MCUPS (millions of updates per second) is around 128×64×200÷50 = 32768.

Table 2 shows the comparison of the memristor-based SOM and current state-of-the-art SOM hardware system.

Table 2 Comparison of the memristor-based SOM and current state-of-the-art SOM hardware system.

Design	Our work	[5]	[6]	[7]	[8]
**Tech.**	Memristor SOM	FPGA AW-SOM	NOC-SOM	FPGA SOM	CMOS SOM
**Vec. Dim.**	128	3	256	3	16
**Map size**	8×8	5×5	16×16	16×16	16×16
**Frequency (MHz)**	200	100	250	100	100
**MCUPS**	32768	~	18597	25344	9102
**Power consumption**	154.8 mW （training）<br>2.56 mW （testing）<br>(For image processing: 5×64 array)	204 mW	~	~	~
**in-situ**	Yes	~	Yes	Yes	No

Supplementary References

1. Fernando, B. R.; Hasan, R.; Taha, M. T. In *Low Power Memristor Crossbar Based Winner Takes All Circuit*, 2018 International Joint Conference on Neural Networks (IJCNN), IEEE, (2018).

2. Chi, J.-H.; Chu, S.-H.; Tsai, T.-H. In *A 1.8-V 12-bit 250-MS/s 25-mW self-calibrated DAC*, 2010 Proceedings of ESSCIRC, IEEE, (2010).

3. Choo, K. D.; Bell, J.; Flynn, M. P. In *27.3 Area-efficient 1GS/s 6b SAR ADC with charge-injection-cell-based DAC*, 2016 IEEE International Solid-State Circuits Conference (ISSCC), IEEE, (2016).

4. Atef, M.; Atef, A.; Abbas, M. In *Low-power transimpedance amplifier for near infrared spectroscopy*, 2016 IEEE International Symposium on Circuits and Systems (ISCAS), IEEE, (2016).

5. Cardarilli, G. C., Di Nunzio, L., Fazzolari, R., Re, M. & Spanò, S. AW-SOM, an algorithm for high-speed learning in hardware self-organizing maps. *IEEE Transactions on Circuits and Systems II: Express Briefs* **67**, 380-384 (2019).

6. Abadi M, Jovanovic S, Khalifa KB, Weber S, Bedoui MH. A scalable and adaptable hardware NoC-based self organizing map. *Microprocessors and Microsystems* **57**, 1-14 (2018).

7. Hikawa H, Maeda Y. Improved learning performance of hardware self-organizing map using a novel neighborhood function. *IEEE transactions on neural networks and learning systems* **26**, 2861-2873 (2015).

8. Ramirez-Agundis A, Gadea-Girones R, Colom-Palero R. A hardware design of a massive-parallel, modular NN-based vector quantizer for real-time video coding. *Microprocessors and Microsystems* **32**, 33-44 (2008).
